# Generation of G51D and 3D mice reveals decreased **α-**synuclein tetramer-monomer ratios promote Parkinson’s disease phenotypes

**DOI:** 10.1038/s41531-024-00662-w

**Published:** 2024-02-29

**Authors:** Silke Nuber, Xiaoqun Zhang, Thomas D. McCaffery, Tim E. Moors, Marie-Alexandre Adom, Wolf N. Hahn, Dylan Martin, Maria Ericsson, Arati Tripathi, Ulf Dettmer, Per Svenningsson, Dennis J. Selkoe

**Affiliations:** 1 Ann Romney Center for Neurologic Diseases, Brigham and Women’s Hospital and Harvard Medical School, Boston, MA 02115 USA; 2 Neuro Svenningsson, Department of Clinical Neuroscience, Karolinska Institutet, 17176 Stockholm, Sweden; 3 Electron Microscopy Laboratory, Department of Cell Biology, Harvard Medical School, Boston, MA 02115 USA

**Keywords:** Parkinson's disease, Parkinson's disease

## Abstract

Mutations in the α-Synuclein (αS) gene promote αS monomer aggregation that causes neurodegeneration in familial Parkinson’s disease (fPD). However, most mouse models expressing single-mutant αS transgenes develop neuronal aggregates very slowly, and few have dopaminergic cell loss, both key characteristics of PD. To accelerate neurotoxic aggregation, we previously generated fPD αS E46K mutant mice with rationally designed triple mutations based on the α-helical repeat motif structure of αS (fPD E46K→3 K). The 3 K variant increased αS membrane association and decreased the physiological tetramer:monomer ratio, causing lipid- and vesicle-rich inclusions and robust tremor-predominant, L-DOPA responsive PD-like phenotypes. Here, we applied an analogous approach to the G51D fPD mutation and its rational amplification (G51D → 3D) to generate mutant mice. In contrast to 3 K mice, G51D and 3D mice accumulate monomers almost exclusively in the cytosol while also showing decreased αS tetramer:monomer ratios. Both 1D and 3D mutant mice gradually accumulate insoluble, higher-molecular weight αS oligomers. Round αS neuronal deposits at 12 mos immunolabel for ubiquitin and pSer129 αS, with limited proteinase K resistance. Both 1D and 3D mice undergo loss of striatal TH+ fibers and midbrain dopaminergic neurons by 12 mos and a bradykinesia responsive to L-DOPA. The 3D αS mice have decreased tetramer:monomer equilibria and recapitulate major features of PD. These fPD G51D and 3D mutant mice should be useful models to study neuronal αS-toxicity associated with bradykinetic motor phenotypes.

## Introduction

Cytoplasmic αS deposits, including Lewy bodies and Lewy neurites, are defining lesions in Parkinson’s disease (PD) brain. A strong genetic association exists between early onset familial forms of PD (fPD) and various αS mutations that are collectively localized in the membrane-binding N-terminal region. In humans (hu), the G51D αS mutation causes an early onset PD which responds to L-DOPA but rapidly progresses to severe phenotypes, including dyskinesias, dementia, limb myoclonus, and seizures^1–3^. Neuropathological examinations showed αS cytoplasmic inclusions in the cerebral cortex and in the brainstem associating with neuronal loss^4^.

Studies of the normal cellular function of αS suggest that it transiently interacts with vesicle membranes, regulating the trafficking and exocytosis of synaptic and other vesicles^5–7^. On the other hand, cell culture experiments indicate that abnormal αS oligomers can bind to and perturb membrane function and contribute to cell death^8–10^. Neuronal culture studies have suggested that the fPD G51D and A30P αS missense mutations have less membrane affinity than the wild-type (wt) protein and may therefore induce cytotoxicity via buffer-soluble oligomers^11^, arguing for a pathogenic role of excessive aggregation-prone monomers independent of their association with membranes .

Understanding the transition of physiological αS to pathogenic oligomers requires knowledge of the normal forms of the protein in healthy neurons. Our group described and characterized a previously unrecognized native form of cellular αS: metastable, helically-folded tetramers of ~58 kDa (4 × 14,502 monomers)^12–15^. Although this concept was initially controversial^16,17^, more than 10 other laboratories also obtained evidence for the existence of physiological tetramers and related conformers^18–23^. Intact-cell crosslinking of freshly biopsied hu control brain and live-cell fluorescence complementation supported αS occurring as both physiological tetramers and monomers, and tetramers are also formed by the closely-related βS and γS proteins^14^. Regarding the relevance of tetramers to disease, we found that all fPD-causing αS missense mutations decrease the physiological tetramer:monomer (T:M) ratio in neurons, thereby increasing the levels of aggregation-prone monomers^14^. The relevance of the tetramers for neuronal physiology is further supported by the recent discovery that human neurons bearing loss-of-function mutations in glucocerebrosidase (GBA1)^22^ or LRRK2^24^ have decreased T:M ratios of their endogenous wt αS protein, and genetic correction or restoration of normal glucosylceramide levels or inhibition of LRRK2 each correct these decreases.

The effect of the E46K mutation on αS tetramerization was exacerbated by strategically introducing two additional homologous E-to-K mutations into the adjacent KTKEGV repeats (at residues 35 and/or 61). Expression of tetramer-lowering 3 K αS (E35K + E46K + E61K) led to formation of membrane rich inclusions in cells and LB-like inclusions in transgenic mice, associated with a L-DOPA-responsive motor syndrome^25^. In accord with a role of tetramerization for αS’s physiological function, the insertion of tetramer-abrogating mutations into 6 imperfectly repeated KTKEGV motifs slowed the kinetics of vesicle exocytosis conferred by WT αS in living neurons and suggested that tetramers participate in normal vesicle trafficking^5,26^.

We recently applied a similar protein engineering method to generate a rationally designed amplification of the G51D mutation into ‘3D’ αS (V40D + G51D + V66D) by inserting G51D-analogous mutations into the immediately adjacent 11-amino acid repeats. Indeed, G51D and more so 3D αS decreased the αS60:αS14 (T:M) ratio, resulting in an excess of soluble (cytosolic) αS compared to WT, causing toxicity in rat primary neurons and in iPSC-derived human neuronal cells^27^. Here, we provide the first characterization of transgenic mice expressing G51D αS and its amplification to 3D αS. We show that both mutations reduced αS T:M ratios, leading to a pathological excess of soluble αS monomers in mouse brain. Both G51D and the 3D αS mice developed a prominent slowness in motor performance beginning at ~6 months (mos) and progressively worsening by 12 mos compared to expression-matched hu WT αS mice, paralleled by a loss of dopamine levels and TH+ neurons in the midbrain. Neuropathologically, G51D and 3D αS mice showed larger-sized somatic inclusions with limited resistance to proteinase K treatment, and these were immunoreactive for pSer129 αS and ubiquitin. Finally, treating symptomatic 3D mice with L-DOPA temporarily reversed the bradykinesia-type motor deficit.

## Results

### Amplifying the fPD G51D αS mutation in vivo shifts αS tetramers toward excess soluble αS monomers and then insoluble oligomers

**Fig. 1:**
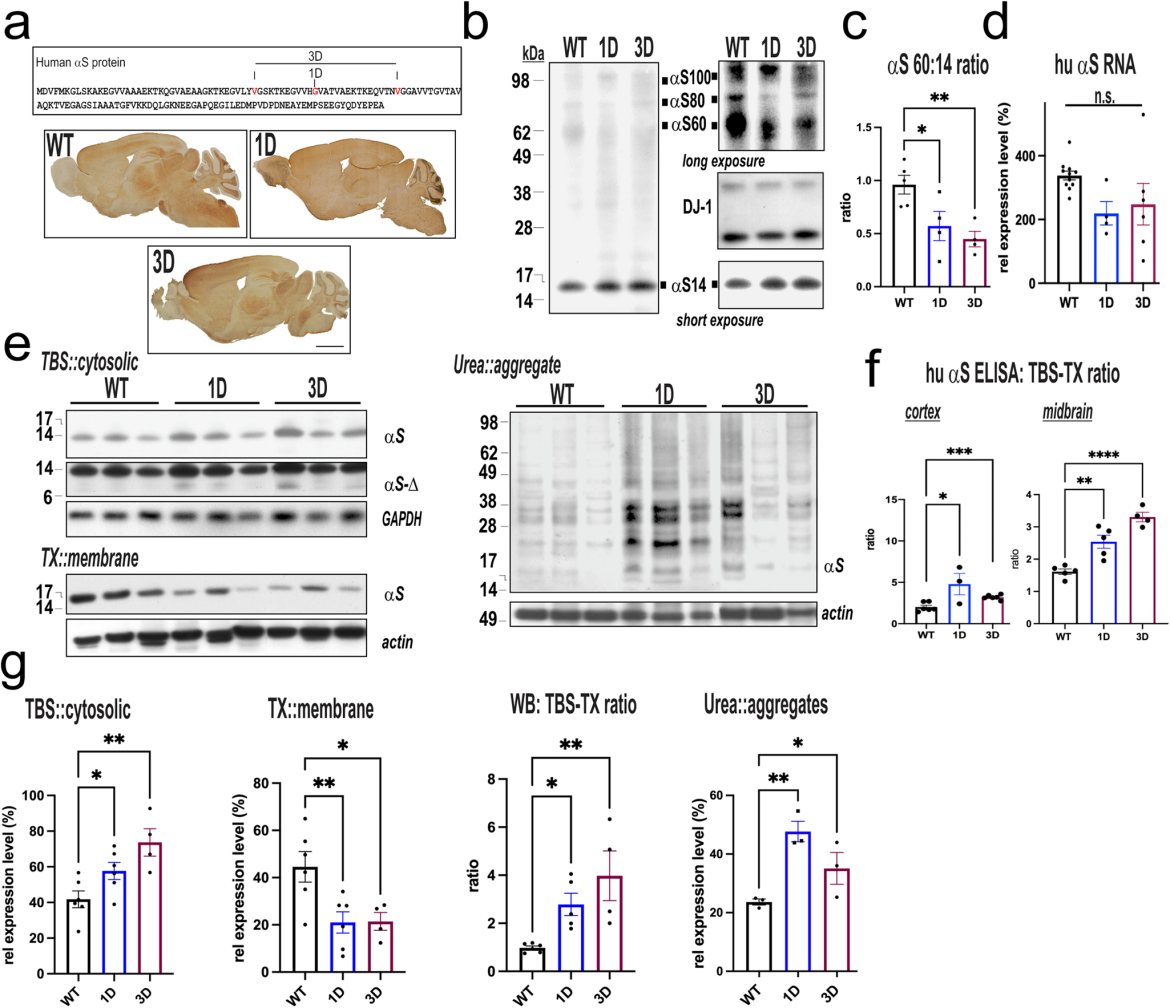
Immunohistochemical and biochemical characterization of 1D and 3D αS mice. **a** Schematic of the amplification of the fPD G51D (1D) αS mutation by replacing hydrophobic amino acids (V,G,V) with three negatively charged aspartic acids (‘3D’) into the adjacent KTKEGV motifs. Panels below show pan-neuronal expression detected by anti hu αS 15G7 antibody of sagittal brain sections from WT, 1D, 3D tg mice. **b** Intact-cell crosslinking of αS in cortical brain bits. Syn1 detects monomeric (αS14) and tetrameric (αS60) αS and probable conformers of the tetramer (αS80, αS100). DJ-1 monomers/dimers serve as control for equal crosslinking and for loading. Longer exposure (right panels) shows a relative decrease in tetramers and an increase in monomers in 1D and in 3D mice. **c** Quantification of WBs like those in (**b**) reveals significant decrease in T:M ratio in D-mutant versus WT (*n* = 4-5 mice per genotype run in 2–3 ind. experiments). **d** RNA expression data for human SNCA (*n* = 4–10 per group). **e** Representative WBs (non-crosslinked) of sequentially extracted TBS-soluble (cytosolic), TX-soluble (membrane) or urea (insoluble) extracts of cortical brain bits. Note the ab 4B12 (epitope spanning aa 103-108) detects monomeric (αS14) and truncations of αS (αS-Δ). GAPDH or actin are loading controls. **f** Quantification of the relative increase in TBS-soluble αS monomers in expression-matched 1D, 3D versus WT and the decrease of TX-soluble membrane fraction associating with presence of higher-molecular αS in the insoluble fraction is quantified from WBs (*n* = 4–6 mice per group, *N* = 2–3 ind. experiments). **g** Hu-specific αS ELISA confirms a significantly higher cytosolic:membrane (TBS/TX) ratio in 1D, 3D versus WT (*n* = 3 ind. experiments). Data are mean ± SEM. One-way ANOVA, post Tukey. *p < 0.05, **p < 0.01, ***p < 0.001, ****p < 0.0001. Scale bar, 5 mm.

Our previous studies showed that different PD-causing αS missense mutations decrease the physiological T:M ratio in neurons^14^ and that adding additional G/V→D mutations in the KTKEGV motifs aggravated the T:M shift and induced αS pathology in neuronal culture^14,15,27^. To examine in vivo the G51D mutation and its biochemically amplified 3D mutation (replacing hydrophobic amino acids V40,G51,V66 in 3 adjacent helix-conferring repeat motifs by negatively charged aspartic acids (D); Fig. 1a) and its relevance to hu PD, we created and performed a comparative analysis of novel 1D (G51D) and 3D αS transgenic mice to our previously generated WT-mouse line. The hu αS is expressed under control of the pan-neuronal Thy1 promoter^25^, resulting in hu αS expression throughout the brain (Fig. 1a, lower panels).

We had described and extensively validated a method to trap the cell-lysis-sensitive tetramers by intact-cell crosslinking of fresh, minced brain tissue bits with the cell-penetrant crosslinker DSG^13,14^. Using intact-cell crosslinking, we assessed whether the 3D variant caused a major decrease in αS tetramers (designated αS60) and related conformers (αS80 and αS100) and increase in monomers (αS14). Similar to our cell culture studies^27^, the G51D (1D) mutation significantly decreased the T:M ratio vs. WT (*p* < 0.05), and this was further decreased by expression-matched 3D (Fig. 1b, c; *p* < 0.01, one-way ANOVA, post Tukey). The decrease in the D-mutant αS T:M ratio was confirmed in a second, but significantly lower-expressing 3D αS line (line #36; designated 3DLow or ‘3DL’) (Supplementary Fig. 1a), also showing a lower T:M ratio (Supplementary Figure 1b, c). Both the 1D and 3D-mouse brain matched expression level of our previously^25^ created WT hu αS expressing line (Fig. 1d). Relative to mouse αS the hu αS expression in cortex (measured per specific ELISA, mean ± SEM, each *n* = 5–6) for WT is 3.6 ± 0.6, for 1D is 2.5 ± 0.6, for 3D is 3.0 ± 1, and for 3DL is 1 ± 0.3. We therefor focused our further analyses on these mouse lines with comparable hu αS expression.

Sequential extractions of cerebral cortex (without crosslinking) and western blotting revealed a shift to more buffer-soluble (cytosolic) αS and less Triton X-solubilized αS (membrane fraction) in 1D and 3D vs. WT cortex (one-way ANOVA, *p* < 0.05; Fig. 1e, g), and this shift was confirmed when using a hu αS sensitive ELISA and in a second brain region (midbrain) relevant for motor coordination (Fig. 1f, see also Fig. 5b). The TX-insoluble membrane pellets were then solubilized in 5% SDS/8 M urea buffer, previously shown to break down αS aggregates in PD brain^28^. The SDS/urea extracts revealed overall higher-molecular weight (HMW) insoluble αS species, ranging between 20 and 40 kDa in D-mutant vs. WT hu αS expressing mice (One-way ANOVA, *p* < 0.05; Fig. 1e, g).

### The tetramer-monomer shift caused by the D mutation is associated with motor slowness resembling bradykinesia features of PD

**Fig. 2:**
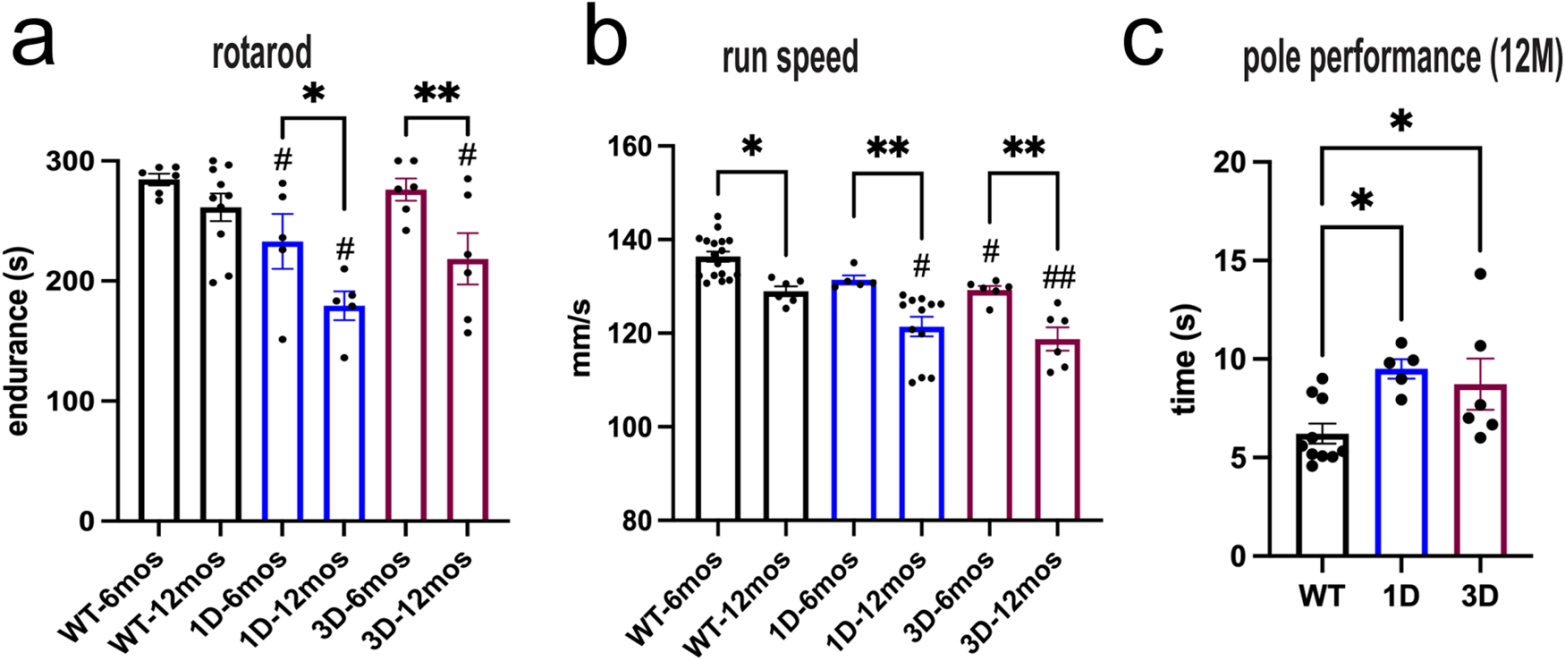
Progressive motor deficits in tetramer-deficient 1D and 3D αS tg mice. **a** Graph quantifies longitudinally motor and balancing skills on a 4–40 rpm accelerating rotarod at age 6 mos and 12 mos. **b** Automated gait scan on a horizontal treadmill shows a more pronounced slowing of movement at 6 mos becoming worse at 12 mos. **c** Graph quantifies ability to turn and climb down a pole at 12 mos. Data are mean ± SEM. *p < 0.05, **p < 0.01, ***p < 0.001; D-mutant vs. WT ^#^*p* < 0.05, ^##^*p* < 0.01; two-way ANOVA (**a**, **b**) or (**c**) one-way ANOVA post Tukey.

Since we previously detected an upstream αS tetramer-loss correlated with robust PD-like motor phenotypes in 3 K αS mice^25^, we subjected the D-mutant mice to motor testing. In general, we observed reduced locomotion 12 mos old D-mutant mice (see Movie S1 and S2). Rotarod testing revealed a reduced ability of D-mutant mice to maintain balance while walking on an accelerating rod at age 6 mos, and this became progressively worse for all D-mut ant mice (1D, 3D) at 12 mos (two-way ANOVA, *p* < 0.05; Fig. 2a; 3DL Supplementary Fig. 1d). No differences in rotarod performance were detected at 3 mos of age (*data not shown*). To further analyze whether this decline was due to deficits in limb coordination or the ability to gain speed during rod acceleration, we photographically analyzed the fine gait patterns of mice running on a horizontal motorized belt (‘GaitScan’; CleversysInc). The analyses showed significantly reduced speed (mm/sec) as early as 6 mos in 3D, and by 12 mos in all D (1D, 3D) mutant mice vs. age-matched WT (Fig. 2b). Gait coupling (homologous/heterologous limb coupling while walking) was unchanged (data not shown). Aged WT hu αS mice also showed subtle impairment in gait scan speed but these were still better compared to the D-mutant mice. In addition, the L-DOPA sensitive^25^ pole test showed significantly impaired performance in expression-matched, 12 mos old 1D and 3D vs. WT mice (Fig. 2c). We previously observed a male-to-female preponderance in motor performances in mice expressing E46K-type (‘3 K’) mutations^25,29^. In general, the motor deficit increased more robustly in the male D-mutant mice (Supplementary Figure 2a-c). Thus, mainly D-mutant females were used to maintain the nascent colony and the histopathological evaluation was conducted in male mice.

### Excess soluble D-mutant αS monomers form somatic inclusions that are phosphorylated but not PK-resistant

**Fig. 3:**
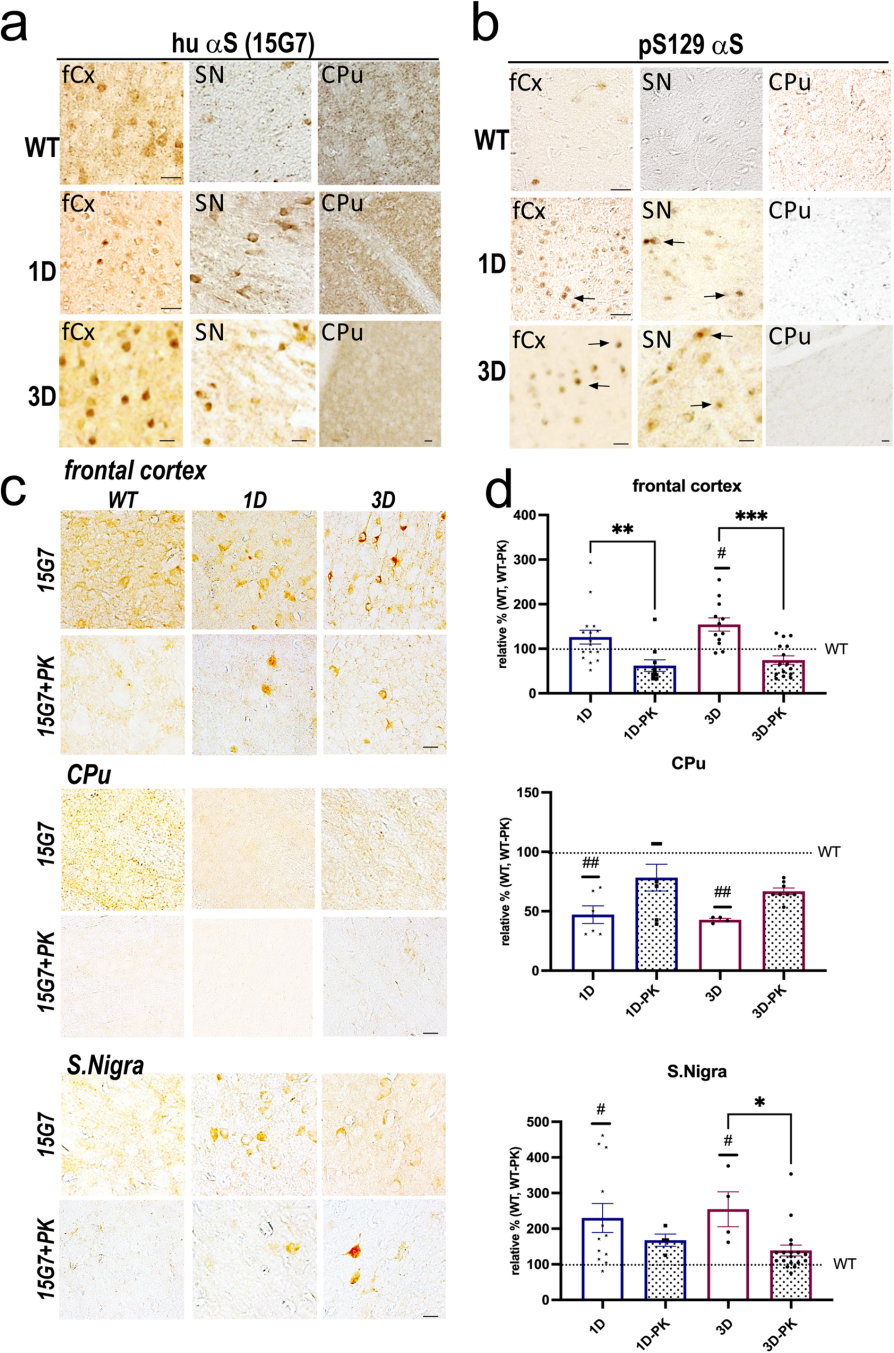
1D and 3D αS forms limited PK-resistant intraneuronal inclusions. **a** Representative high-magnification images of the hu αS signal of total (15G7) αS and **b** pS129+ in the frontal cortex (fCx), midbrain region -- containing the substantia nigra (SN) -- and the caudate-putamen (CPu) of 12 mos old 1D and 3D and expression-matched WT αS mice. **c** Adjacent sections were additionally treated with proteinase K (PK) and **d** the integrated optic densities with or without PK treatment quantified as relative % against WT or WT-PK immunoreactivity. Data are mean ± SEM. Two-way ANOVA, post Tukey. PK-treatment: *p < 0.05, **p < 0.01, ***p < 0.001; D-mutant vs. WT ^#^*p* < 0.05; ^##^*p* < 0.01. Scale bars, 25 µm.

To assess neuropathological changes associated with the shift of tetramers to excess buffer-soluble monomers, and a certain amount of insoluble oligomers (Fig. 1) and accompanying progressive motor deficits (Fig. 2), we searched for αS inclusions in cryostat sections of frontal cortex (fCx), striatum, and substantia nigra (SN)/midbrain in 12 mos old 1D and 3D mice. Total hu αS detected with mab15G7 (hu specific) showed large round inclusions in neuronal somata (Fig. 3a). However, a more diffuse and small-punctate immunoreactivity of neurite fibers was detected in D mutant vs. WT αS mice in the fiber-rich striatum (CPu) (Fig. 3a, right panels). An antibody against pS129+, a standard marker of LB aggregates in hu PD/DLB, also highlighted round αS inclusions in neuronal soma, often situated near the nucleus in the fCx and SN (Fig. 3b left and middle panel). Almost no pS129+ immunoreactivity was detected in the CPu (Fig. 3b, right panels). In some neurons, the perinuclear pS129 inclusions overlapped with the nuclear area (arrows, Fig. 3b). However, ultrastructural analyses did not substantiate nuclear aggregates in cortex or midbrain *(data not shown)*. We next probed sections for proteinase K (PK) resistance, a common feature of hu LBs, that, however, may not represent all toxic (oligomeric) αS species^30^. Occasionally, PK-resistant inclusions were detected in fCx and SN/midbrain of 1D and 3D mice (Fig. 3c, middle panels). Statistical evaluation of the densities (integrated optic densities; IOD) revealed most 1D and 3D αS inclusions decreased significantly after PK digestion (two-way ANOVA, *p* < 0.05), suggesting that PK-resistance is not a prominent feature of G51D or 3D αS aggregates. This result was substantiated in the hippocampus (Supplementary Fig. 3a, b), a region vulnerable to Thy1-directed hu αS overexpression^31^. The overall (non-digested and PK-digested) striatal αS staining was less in D-mutant vs. WT hu αS brain sections, and only background staining was observed in the simultaneously PK-processed WT hu αS mouse brain sections (Fig. 3c, d).

### αS 1D and 3D monomers accumulate at cell bodies, forming ubiquitin and pS129+ deposits

**Fig. 4:**
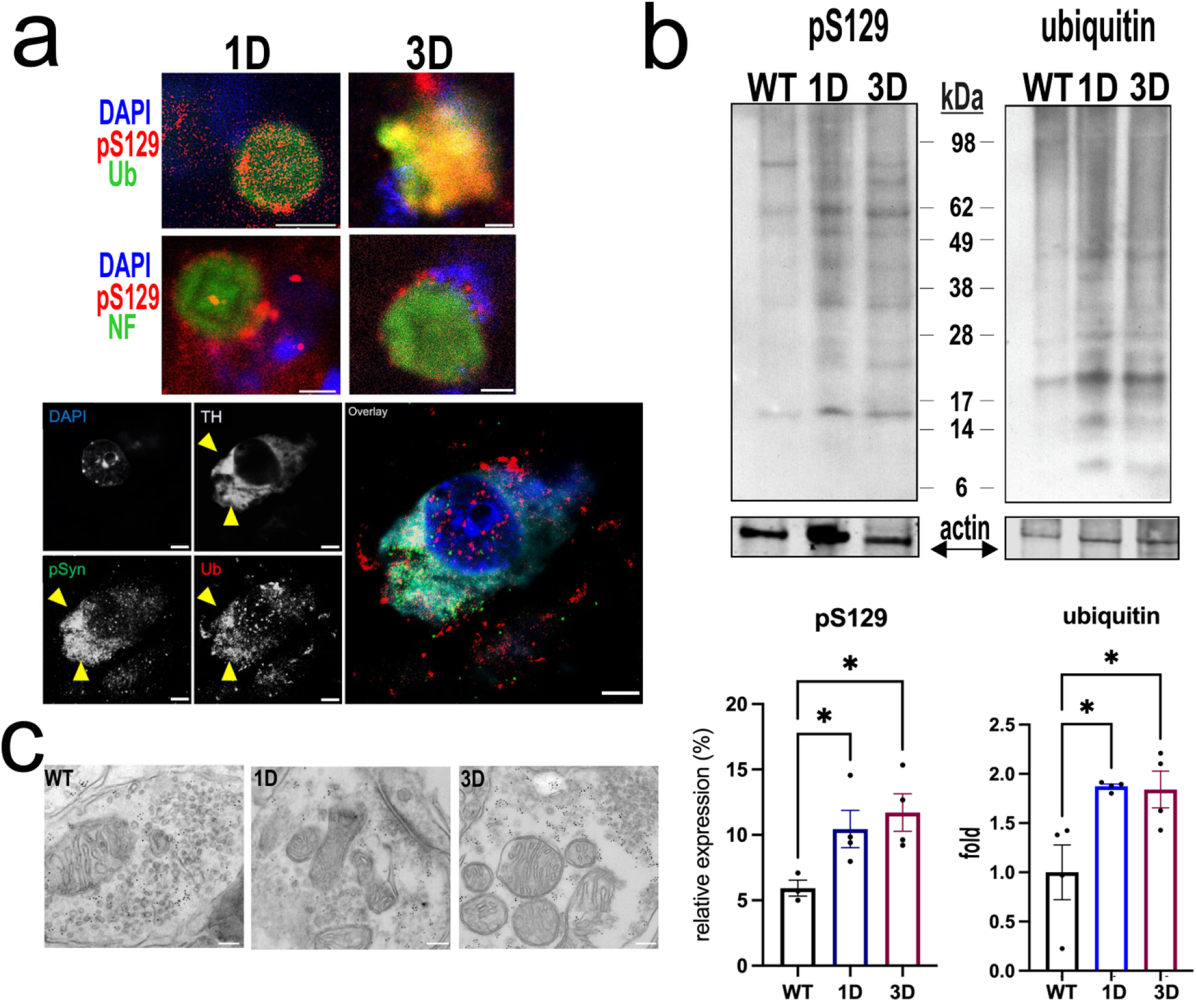
Midbrain DAergic αS inclusions in 1D and 3D mice show features of hu LBs. **a** Perinuclear pSer129+ inclusion bodies in midbrain neurons are co-immunolabeled for ubiquitin (Ub) (upper panels) and surrounding neurofilament (NF)+ profiles, lower panels are high magnifications of TH+ neurons displaying pSer+ and Ub+ inclusions. **b** Representative blots for pS129 and ubiquitin of urea-extracts from midbrain of 1D, 3D vs. WT mice. Data are mean ± SEM. One-way ANOVA, post Tukey. *p < 0.05. **c** Representative EM images revealed finely dispersed αS-immunogold in WT synapses (left), fewer αS + puncta in 1D (middle) or 3D striatal synapses (right) in 12 mos mice. Scale bars **a**: 2 µm; **b**: 5 µm; **c** 100 nm.

αS mutations that confer less membrane association (e.g. A30P) have been shown to develop deposits having certain posttranslational modifications, including pS129+ and ubiquitin+, in mouse models^32–34^. These are also features of LBs in the hu G51D PD brain^35^. To further characterize the somatic inclusions in midbrain dopaminergic neurons, we immunostained sections for TH, pS129, and ubiquitin. Notably, we found larger-sized aggregates within TH+ neurons in 1D and 3D mice, that consistently co-stained for pS129 and ubiquitin (Fig. 4a). Interestingly, the aggregates were surrounded by neurofilament (NF) labeling, a marker that often associates with pS129+ LBs in DLB and PD^36^ (Fig. 4a). We did not find evidence for such inclusions in age- and expression-matched hu WT αS DAergic neurons (*data not shown*).

To quantify these results, we conducted sequential protein extractions of midbrain tissue pieces and probed for pS129 and ubiquitin in the aggregate-rich urea-fraction by WB. Midbrain insoluble proteins showed that both pS129 and ubiquitin signals were elevated in 1D and 3D vs. WT mouse brain (One-way ANOVA, *p* < 0.05), further substantiating pathologic αS aggregation in D-mutant mice (Fig. 4b).

To explore the ultrastructure of the D-mutant αS deposits further, we performed immunogold EM in SN of 1D, 3D and hu WT αS mice at 12 mos. In the 1D and 3D mice, immunogold-reactivity was less frequent at synaptic vesicles when compared to WT neurons, which exhibited more finely distributed αS around synaptic vesicles (Fig. 4c).

### D-mutant αS mice have dopaminergic neurodegeneration, and the associated motor deficits are partly responsive to L-DOPA

**Fig. 5:**
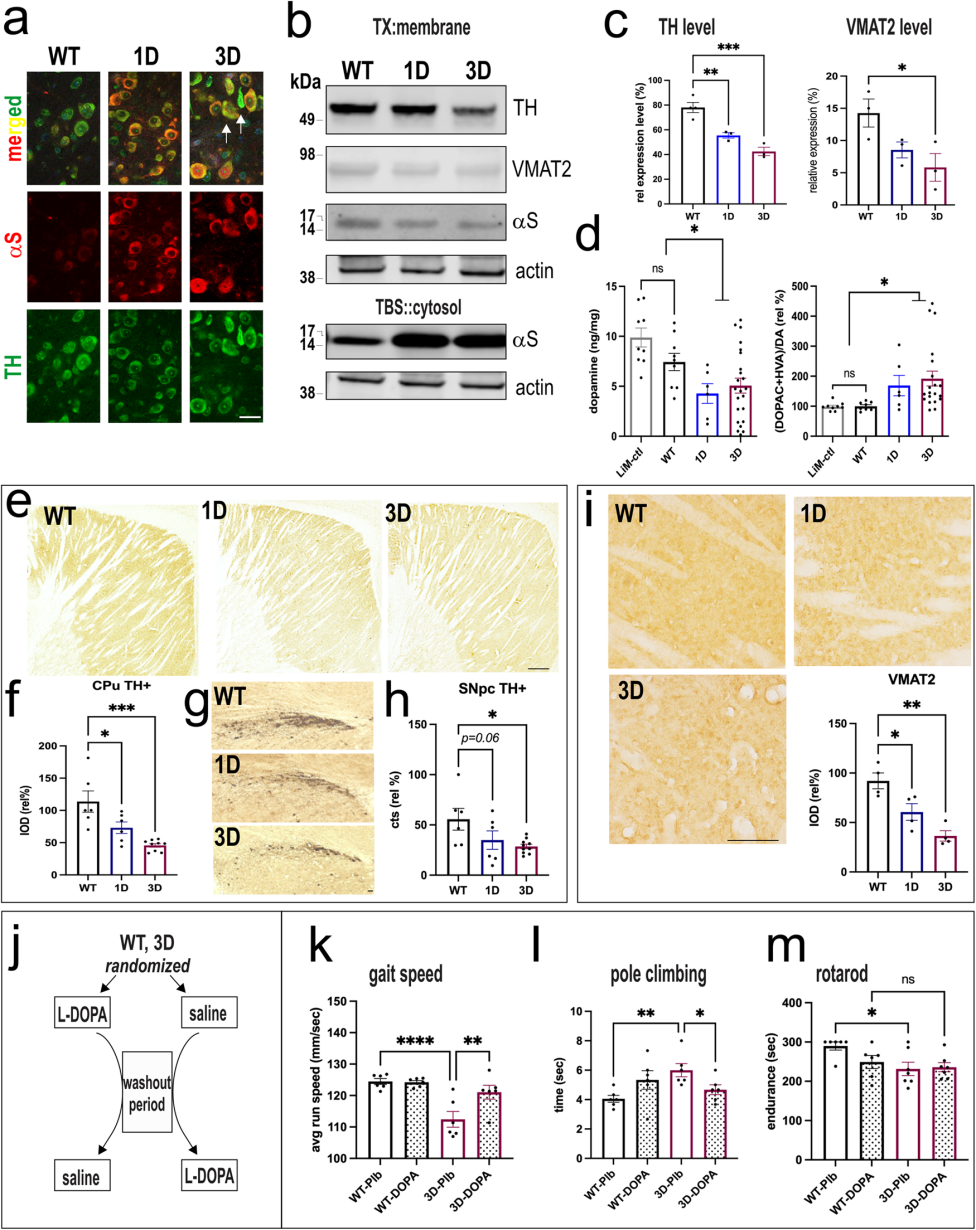
Dopaminergic neurodegeneration in D-mutant αS mice. **a** Representative high-magnification confocal images of αS (15G7) colocalization in DAergic neurons of D-mutant αS mice showed prominent somatic hu αS accumulations in TH-positive nigral neurons. Occasionally deformed or pycnotic neurons (*arrows*) were detected in 3D brain sections. **b** WBs of DAergic proteins and hu αS in midbrain of 12 mos D-mutant and WT mice and **c** quantification of relative TH, VMAT2 level or **d** HPLC assay of striatal dopamine (DA) and dopamine metabolite (DOPAC+ homovanillic acid) turnover in 12 mos mice. **e** Rep. images of tyrosine hydroxylase (TH) positive striatal fibers and **f** relative optical density and **g** relative % of neuronal counts, quantified in **h** (avg. scans of 4 sections of *n* = 3 mice/group). **i** Rep. images of VMAT2 immunoreactivity in striatal fibers and relative optical density (avg. scans of 4 sections of n = 3 mice/group). **j** Pharmacological rescue of motor deficits of symptomatic 3D mice by L-DOPA (12.5 mg/kg) in a cross-over trial: **k** increase in gait speed on a horizontal treadmill (GaitScan) and **l** pole climbing. **m** The single L-DOPA application did not rescue deficits on the challenging rotarod. Data are mean ± SEM. One or two-way ANOVA, post Tukey, *p < 0.05; **p < 0.01, ***p < 0.001, ****p < 0.0001. Scale bar **a**, **g** 25 µm **f** 500 µm, **i** 100 µm.

We next assessed whether the pathological accumulation of 1D and 3D αS monomers at DAergic cell somata and the associated motor phenotypes correlated with DAergic neuron degeneration. The quantification of tyrosine hydroxylase (TH+) neurons was conducted in male mice, since subtle differences in sex hormones can contribute to variances between male and female mice in dopamine neurochemistry^37^. Co-staining showed a clear-cut overlap of hu (total) αS in midbrain DAergic (TH+) neurons of D-mutant mice and occasionally deformed/pycnotic neurons (Fig. 5a, arrows).

We measured significant reduction in the TH+ level in SN protein extracts in 12 mos old 1D and 3D αS mutant mice compared to WT hu αS mice (Fig. 5b, c).

In addition to TH, the vesicular monoamine transporter 2 (VMAT-2), a DAergic vesicle marker reduced in PD^38^, showed significantly less signal (*p* < 0.05) (Fig. 5b, c), further substantiating DAergic loss in D-mutant mice. Probing for hu αS in the midbrain SN region substantiated the expected excess of TBS buffer-soluble αS (Fig. 5b; for cortex, see Fig. 1e-g). In addition to the loss of TH and VMAT2, expression-matched 1D and 3D mice displayed a significant decrease in DA measured by HPLC (one-way ANOVA, Tukey; *p* < 0.05 vs. Ntg and WT control mice) (Fig. 5d). The turnover rate of DA was increased, as measured via the metabolites 3,4-Dihdroxyphenylacetic acid (DOPAC) and homovanillic acid (HVA) (Fig. 5d right panel, one-way ANOVA, Tukey *p* < 0.05 vs. Ntg control littermate or WT); this might be similar to hu PD, which shows an increase in DA turnover correlated with VMAT2 loss^38^. Interestingly, we noticed that D-mutant mice displayed darker coat (fewer grays) at both at 6 and 12 mos vs. WT (Supplementary Figure 4). Dopamine (DA) agonists have been reported to inhibit darkening of coat color in Agouti mice^39^, suggesting decreases in DA metabolism may associate with the relative darkening of fur we observed in D-mutant mice.

To confirm that decreased striatal DA was associated with DAergic fiber degeneration, we quantified tyrosine hydroxylase (TH) and VMAT2 immunoreactive axons and their terminals (Fig. 5e, i). We analyzed the dorsal striatum, which is rich in projections from the DAergic neurons of the substantia nigra pars compacta (SNpc). Here, we measured a ~25% reduction in the density of TH+ fibers in in 12 mo old 1D, and a ~50% reduction in 3D compared to WT hu αS mice (Fig. 5e, f) (one-way ANOVA, *p* < 0.05). Since nerve fiber degeneration may precede DAergic cell loss in PD^40^, we next counted DAergic neurons in the SNpc to estimate the relative percentage neuronal loss (Fig. 5g). We found a ~15% reduction of TH+ SNpc neurons in 3D (*p* = 0.01) and a strong trend of reduced TH+counts (*p* = 0.06) in 1D vs. WT mice. Since αS can regulate TH^41,42^, we analyzed additional striatal sections for VMAT2. The VMAT2 immunoreactivity in the dorsal striatum fo D-mutant mice was −34% ±18% for 1D and −60% ±11% for 3D, further substantiating degeneration of the dopaminergic neuropil (Fig. 5i).

Given the numerous phenotypic parallels of the D-mutant mice to PD, we analyzed the effects of L-DOPA on the more strongly impaired 3D mice, using a blinded, randomized cross-over trial (Fig. 5j). We used a relatively low dose of L-DOPA previously published to ameliorate a moderate DAergic phenotype and to avoid induction of stereotypic behaviors in mice [e.g^43^.] and that we previously showed improved gait (limb coupling) and pole climbing behavior in 3K mice^25^. A single L-DOPA injection significantly improved the gait speed (Fig. 5k) and pole test performance of the symptomatic 3D mice, as measured by a shorter time to climb down the pole (Fig. 5l: 3D-Plb vs. 3D-DOPA, two-way ANOVA, *p* < 0.05). However, L-DOPA did not improve the highly abnormal 3D performance on the rotarod (Fig. 5m), suggesting a contribution from the cortical pathology to this motor impairment.

## Discussion

αS constitutes the major component of Lewy bodies and its causal contribution to the etiology of PD is evident. αS can adopt physiologic multimeric^12^ or pathologic oligomeric and fibrillar conformation states, but it is still debated as to which of the different conformational states of αS mediate neurotoxicity. The present study was motivated by recent evidence showing that rationally designed E→K mutations in the KTKEGV motifs display a dramatically reduced propensity to form physiologic tetramers and instead develop misfolded αS inclusions in culture^14,15^, and that these variants triggered aggregation, DAergic and other neuronal pathologies and robust PD phenotypes in mice^25^. These results were interpreted as a confirmation of the ‘tetramer hypothesis’ in the sense that tetramer loss promoted the more strongly phospholipid membrane associated αS monomers (given the positive charges of the E→K mutations), that can accumulate into toxic and LB-type aggregates over time^25^.

Here we have made transgenic mouse lines that comprise the G51D αS singly mutant mouse using the neuron-specific Thy1 promoter or mice expressing and engineered amplification of the G51D mutation. These pan-neuronally expressing 1D and 3D mice display a loss of physiological tetramers and produce high levels of buffer-soluble (cytosolic) αS monomers, far exceeding those of an expression-matched WT αS mouse line. Mouse models with additive E→K mutation demonstrated that enhanced tetramer-abrogation increased pathologic excess monomers in the membrane-fraction (TX-100 soluble extract) and accelerated αS deposition in vesicle and lipid-rich round inclusions^25^, resembling pale bodies and vesicle-rich LBs in PD^44^. Now we show that the excess soluble monomers (TBS-soluble) in the fPD 1D and combined 3D mutations also lead to the accrual of buffer insoluble higher-molecular weight, apparently dimeric and trimeric αS species in cortical and midbrain neurons. As a consequence, 1D and 3D mice develop pS129+ and ubiquitinated inclusions with limited PK-resistance that induce DA loss, neurodegeneration and a L-DOPA responsive bradykinesia-like motor deficit, fundamental characteristics of PD that were lacking in most previously reported singly-mutant or hu wild-type αS overexpressing mice.

Our data suggest a mechanistic route that potentially contributes to neuropathology and motor symptoms in PD based on the perturbed assembly of the αS tetramers as a consequence of destabilizing mutations in αS. The rational biochemical amplifications were initially exemplified in the 3 K mouse having severe PD-like resting tremor, uniquely among reported αS model mice^25^. Mechanistically, the 1D and 3D mutant mice further support our hypothesis that the reason this new mouse model closely mirrors some key features of hu PD is that the underlying event – an altered tetramer-monomer equilibrium – may occur in PD, DLB and other human synucleinopathies. Genetic and experimental evidence for this concept comes from hu fPD αS missense mutations^14^ or αS triplication^45^, GBA deficiency^22^ and hu LRRK2 mutations^24^ in patient-derived neurons. In addition, the environmental PD-associated toxin paraquat also decreased normal αS tetramers, leading to an excess of calpain-truncated monomers and inducing toxicity in primary mouse neurons^46^. The probable molecular mechanism underlying D, K and other tetramer-reducing mutations likely is the alteration of proper transient formation of the well-documented amphipathic helix that requires physiological membrane tethering observed by WT αS^47^. Previous experimental studies reported that G51D αS has a lower affinity for binding to negatively charged vesicles than WT, likely via decreased amphipathic helix formation^48^. Thus, reversible αS amphipathic helix formation and dynamic multimerization appear to regulate normal membrane-tethering function of αS, and abrogating physiological multimers interferes with this function and has pathogenic consequences in vivo.

Correlations between motor disabilities and toxic aggregates were assessed in 3 brain regions of 12 mos old, symptomatic D-mutant mice. Previous studies showed that G51D αS was found highly phosphorylated at Ser129 in primary culture^48^ and in hu G51D PD brain^2,35^. Similarly, G51D and 3D mutant mice displayed large round perinuclear pSer129+ inclusions. Although the G→D mutations initially led to excess soluble (cytosolic) monomers, we found that the 1D and 3D mutation eventually led to some insoluble higher-molecular species (oligomers), ubiquitin and pSer129+ deposits in TH+ neurons that degenerated. Most of these inclusions did not display resistance to in sito digestion by proteinase K (PK) in cryostat sections. The composition of PK-resistant aggregates is incompletely defined but evidence suggests that they consist of at least some fibrillar αS^49–51^ and/or contain lipid droplets^52^. In contrast, small sized oligomers, and non-PK-resistant inclusions are detected in our G51D and 3D mutant mice. In support of this observation are two recent studies that showed a distinct polymorphism of G51D fibrils, leading to smaller sized oligomers with decreased PK stability but robust neuropathology when compared to all other αS mutations^53,54^. In these studies, A30P αS, although it had a similar decrease in membrane association, displayed less neurotoxicity in SH-SY5Y DAergic neuron culture than G51D^54^. This difference in DAergic toxicity might explain DA loss detected in fPD G51D αS mice of our study, which was not reported in A30P αS mice^32^.

Expressing the hu G51D and 3D mutations produced DAergic deficiency and a bradykinetic gait impairment partially responsive to L-DOPA, which are similar to our observation in tetramer-abrogating 3 K mice^25^ and make the D-mutant mice a useful model for mechanistic studies and therapeutic screening.

We found that TH, VMAT2 and pSer129 were significantly reduced in nigrostriatal tissue when compared to WT mice (Figs. 3–5). Likewise, DA level were reduced associating with an increase in DA turnover, an early compensatory mechanism of vulnerable DAergic neurons in hu PD. Denervation within the nigrostriatal pathway decreases VMAT2 availability in PD brain^55^ and this is consistent with our previous findings that overexpressing G51D and 3D mutant αS in cultured neurons had prominent adverse effects on neuronal fiber integrity^27^. The unexpected decrease of pSer129 in the striatum of D-mutant vs. WT mice can be explained by this decrease in nerve fibers. Very recent studies suggest that a portion of pSer129+ can be physiologically involved in positively regulating excitatory synaptic activity^56^; thus the relative decrease of pSer129 might contribute to reduced synaptic and thus locomotor activity, and L-DOPA treatment of 3D mutant mice temporarily compensates a part of these deficits. Interestingly, the fur coat seemed somewhat darkened in D-mutant vs. WT mice. Black fur color was positively correlated with a decrease in brain DA signaling of Agouti mice^39^ and might be an interesting biomarker for evaluating changes in DA metabolism in D-mutant mice in future experiments.

The results leading to the conclusion of this study were obtained in Thy1-G51D and 3D-mouse lines, with Thy1 promoter specific overexpression predominantly in cortical and to a lesser extent in subcortical regions. Our results will need to be corroborated in the context of either humanized or a knock-in models and/or in mice expressing G51D under cell-type specific promoters. However, introducing G51D mutation (by CRISPR/Cas9) into the genome confirmed mislocalization of the mutant αS and pS129+ in cortical synapes^57^ and DAergic deficits in rats^58^, substantiating our findings of increased αS monomer solubility and phenotypes in mice. A 2^nd^ limitation of our study is, that it did not reveal a significant increase in pathologic phenotypes between 1D and 3D mutant mice when compared to 3 K > 1 K > WT^25^. A plausible explanation is a gradual increase in membrane association by K-mutant αS (due to the increasing positive charge, which enhances αS membrane binding) vs. adverse membrane affinities in the negative-charge enriched D mutant series, where the membrane repellant properties of G51D, making it more cytosolic, cannot be further increased by additional insertions of D mutations. This difference in solubility by these mutations may induce different mechanisms downstream of the tetramer-loss. Finally, this study focused on cortex, midbrain and striatum, but multiple brain regions, including the spinal cord, could have contributed to the motor abnormalities.

The study presented here adds to an emerging support that perturbation of the αS tetramer stability may contribute to a range of PD disorders, including LRRK2^24^, GBA1^22^, PD/DLB^14,25,27,59^ and paraquat-toxicity^46^. The mechanisms by which specific mutations in different gene products cause similar synucleinopathies remain intriguing and needs to be further clarified. The therapeutic implications of the recapitulation of PD phenotypes in our 1D and 3D mice include screening for small molecules that can stabilize physiological tetramers or decrease the levels of oligomerization and neurotoxic aggregates. We previously showed the female sex hormone estradiol^29^, decreasing lipid desaturation^60–63^, or overexpression of lipid-modifying enzyme GCase1^64^ can each increase in part of the physiological tetramer to monomer homeostasis, and this associated with improved motor phenotypes in 3 K mice. Applying such treatments to D-mutant mice having excess of soluble αS monomers could help support future clinical benefit for PD/DLB patients. αS tetramer stabilization might be analogous to the action of the approved drug tafamidis which stabilizes transthyretin tetramers in patients with that fatal amyloidosis^65^. Our novel fPD G51D mutant mice can be used to explore the differential biochemical and synaptic mechanism of tetramer-abrogation by fPD E46K vs. fPD G51D mutants as regards biochemical differences in αS monomer solubility, that nevertheless gradually converge into shared end-stage LB-type brain pathologies in fPD patients with either mutation.

## Methods

### Generation of D-mutant mice and their treatment

Transgene expression in pThy1 is controlled by the Thy1.2 neuronal regulator. To generate Thy1-G51D and Thy1 3D αS tg mice, the full-length human WT αS cDNA was ligated into pTS (2) vector^66^, and mutagenesis^14^ was used to generate the 1D and 3D variants. Briefly, pThy1-WT-αS, pThy1-1D-αS and pThy1-3D-αS plasmids were generated by XhoI restriction enzyme (New England Biolabs, Ipswich, MA) and digestion of pThy1/Flpbow3 (Addgene plasmid #45181, gift of Dr. Joshua Sanes)^67,68^ was followed by insertion of the respective *SNCA* gene variant by In-Fusion cloning (Clontech, Mountain View, CA). The WT or D-mutant cDNAs were micro-injected into C57BL/6J one-cell embryos. Founder animals were identified by PCR of DNA from ear biopsies using primers specific for the Thy1 promoter and the transgene construct (Thy1-F: 5′-tctgagtggcaaaggaccttagg-3′, Syn-R: 5′-gtggggctccttcttcatt-3′). Out of 5–6 potential founders per line, we established one 3D line with low overexpression (similar level to mouse endogenous αS); one 3D lines (#35) with abundant overexpression (~4 fold) one 3D line (#37) with moderately high overexpression (~2–3 fold) similar to the previously published WT line with a moderately high overexpression (2–3 fold). The 3DL+/+ total hu αS expression level is ~20% compared to either WT, 1D or 3D αS. We chose to focus our characterization on 1D, 3D line #37, which has moderate overexpression at a similar level to our WT line to examine the effects of tetramer abrogation. We plan to make this unique new 1D and 3D model available to the research community (through JAX lab) to pursue PD/DLB mechanisms and drug discovery.

All mice were bred to the C57BL/6J background, were viable and displayed similar expression levels in males and females. Mice of all lines bred successfully, albeit generation of 3D-mouse lines were only successful in the 5th trial, given that each time pups died at P0-P5 (unknown reasons). All mice were kept in normal 12 h light/12 h dark cycles and had free access to food and water. *For L-DOPA treatment*: mice received benserazide (Sigma, 12.5 mg/kg, IP) 20 min prior to L-DOPA (Sigma, 12.5 mg/kg, IP) and the L-DOPA 10 min prior to the start of behavioral testing in a randomized, blinded cross-over design. Behavioral studies were conducted at the Hale-BTM. All animal procedures were approved by the Institutional Animal Care and Use Committee at BWH (IACUC protocol #N000314).

### Intact-cell crosslinking of brain tissue

Dissected brain regions were gently minced into small bits with a razor blade, and the brain bits were washed free of released cytosol and re-suspended in PBS with EDTA-free Complete protease inhibitors (Roche Applied Science). Intact-cell crosslinking was then conducted with minor modifications of our established protocol^13^. Briefly, the cell-permeable crosslinker DSG was prepared at 1 mM final concentration in DMSO immediately before use. Samples were incubated with crosslinker for 40 min at 37 °C with rotation. The reaction was quenched by adding Tris, pH 7.6, at 100 mM final concentration and incubated for 10 min at RT. After quenching and aspiration of the supernatant, proteins in intact tissue were extracted directly in TBS/1% Triton X-100 and then subjected to western blot analyses (see below).

### Sequential tissue extractions

The regional expression pattern of αS was initially examined at age 6 mo. Mice were anesthetized, decapitated and the brains dissected on a chilled stage. For sequential extractions tissues were homogenized in 2 volumes of TBS+ [50 mM Tris-HCl, pH 7.4, 175 mM NaCl; 5 mM EDTA; protease inhibitor cocktail (Calbiochem, CA)] and spun for 30 min at 120,000 × *g*. The pellet was subsequently extracted in TBS+ containing 1% Triton X-100, then in TBS+ containing 1 M sucrose, The TX-insoluble pellet was then extracted in RIPA buffer (TBS + , 1% NP-40, 0.5% sodium deoxycholate, 0.1% sodium dodecyl sulfate), with each extraction step followed by ultracentrifugation for 30 min at 120,000 × *g*. Final pellet was solubilized in 8 M Urea/5% SDS.

### Western blot analyses

For Western blotting, 10–25 μg total protein of sequential extracts of dissected mouse brain regions were electroblotted onto nitrocellulose membranes (Millipore, Bedford, MA). For improved immunodetection of αS (monomers of which are prone to washing off filters^69,70^), the membranes were fixed in 0.4% paraformaldehyde (PFA) for 20 min. If no higher-molecular signal was expected, blots were cut prior blocking allowing simultaneous incubation with antibodies against the target protein and the loading control of the same experiment (Figs. 1e and 5b). After washing in tris-buffered saline (TBS), membranes were blocked for 1 h at RT in TBST (tris-buffered saline with 0.2% Tween-20) containing 5% bovine serum albumin (BSA). Blots were then incubated with human-specific αS antibody (ab) (15G7, Enzo; 1:500; 4B12, 1:2000; Sigma), ab against phosphorylated (ser129) αS (AB51253; 1:5000; Abcam), TH (AB152; 1:1000; Millipore), ubiquitin (AB 10201-2; 1:1000; Abcam) or VMAT2 (GTX89638, 1:2000; GeneTex) in TBST containing 5% BSA overnight. After washing with TBST, membranes were probed with appropriate secondary antibodies (1:5000, American Qualex, CA), visualized with enhanced chemiluminescence (ECL, PerkinElmer, Boston, MA), and analyzed with the LI-cor gel imaging system. Proteins were normalized to β-actin (A5441, Sigma; 1:3000) or GAPDH (AM4300, Invitrogen; 1:20,000) used as a loading control. All blots were processed in parallel and derived from the same experiment.

### High pressure liquid chromatography

HPLC was conducted as previously described,^71^. To estimate striatal monoamine levels at age 12 mos in WT (*n* = 9), 1D (*n* = 6) or 3D αS tg (*n* = 23) and non-tg littermate (Li-Ntg) control mice (*n* = 9), the mice were deeply anesthetized by CO_2_, quickly decapitated, and the striata dissected on ice, homogenized in 0.1 M perchloric acid, centrifuged, filtered, and stored at −80 °C until analysis for monoamine content. Standard solutions of dopamine hydrochloride, 3,4-dihydroxyphenylacetic acid (DOPAC) and homovanillic acid (HVA) were prepared in 0.1 M perchloric acid to obtain final standard concentrations of 200, 100, 50, 10, 5, 2 and 1 ng/ml. Calibration curves were obtained with the Chromeleon software through linear regression of peak area versus concentration. The analysis was performed on HPLC-ECD system (Dionex Ultimate 3000, ThermoFisher Scientific, Waltham, MA, USA). The separation was performed on a C18 reversed-phase column at 30 °C. The mobile phase (75 mM monobasic sodium phosphate, 2.2 mM OSA, 100 µL/L TEA, 25 µM EDTA, and 10% acetonitrile (*v/v*), pH 3.0), was pumped at a flow rate of 0.4 mL/min. The first and second analytical cells were set to −100 mV and +300 mV, respectively. Processed samples were thawed on ice about an hour before analysis, placed in the autosampler, and kept at 5 °C before injection. Chromatograms were acquired with Dionex Chromeleon 7 software over an acquisition time of 55 min. Analyte concentrations in tissue samples were expressed as ng/mg of frozen tissue.

### RNA

Total RNA samples were isolated from cortical brain bits using mirVana miRNA Isolation Kit (AM1561), and RNA concentrations were determined by Nanodrop. The samples were converted into cDNA using Applied Biosystems High-capacity cDNA reverse transcription kit (#4368813). Afterwards, the cDNA was mixed with Taqman Fast Universal PCR Mix (#4352042) for qPCR analysis using Taqman Gene Expression primers for human SNCA (Hs00240906_m1).

### Electron microscopy

Mice underwent transcardiac perfusion with 4% PFA and the dissected brains were fixed in 4% PFA for another 48 h, subsequently in 2% PFA for 24 h, and then transferred into 0.5% PFA. Dissected brain regions were quenched with 0.2 M glycine in PBS for 10-15 min at RT and then permeabilized by washing for several times in PBS containing 0.1% Triton X-100 (PBST), then blocked for 30 min in 1% (w/v) bovine serum albumin in PBST at RT. A rat antihuman αS antibody (15G7,1:50, Enzo) in PBST containing 1% BSA was added to sections and incubated overnight at 4 °C. Sections were washed five times for 10 min in PBS followed by incubation with rabbit anti-rat bridging antibody (AB 6703;1:100; Abcam) in PBS containing 1% BSA for 1 h at room temperature, then washed five times in PBS, followed by incubation with 15 nm Protein A-gold particles (1:50, Utrecht University Med Ctr) in PBS containing 1% BSA for an hour at room temperature. Sections were subsequently washed in PBS and then fixed in 1% (v/v) glutaraldehyde in PBS for 30 min. For Epon embedding, slices were incubated in 0.5% (w/v) osmium in ddH_2_O for 30 min, washed three times in ddH_2_O then stepwise dehydrated (each step for 10 min) in 70% (v/v) ethanol, 95% (v/v) ethanol and twice in 100% (v/v) ethanol. The slices were incubated in propyleneoxide, infiltrated in 1:1 propylenoxide/TAAB Epon (TAAB Laboratories Equipment Ltd, https://taab.co.uk) and polymerized at 60 °C for 48 h. Each block was cut into 60 nm ultrathin sections using a Reichert Ultracut-S microtome. Sections were placed onto copper grids and stained with uranyl acetate and lead citrate. The sections were examined using a JEOL 1200EX transmission electron microscope. Images were recorded with an AMT 2k CCD camera at 20,000–×30,000 magnification.

### Immunohistochemistry

Mice were sacrificed with an overdose isoflurane, followed by intracardiac perfusion with PBS and ice-cold 4% (w/v) PFA in PBS (pH 7.4). The brain was dissected from the skull and post-fixed in 4% PFA for another 48 h at 4°C. Brains were cut into 25 µm cryotome sections and immunostained. Briefly, after treatment with H_2_0_2_ (3% in PBS, 20 min), optionally proteinase K (P4850, 0.01% in 5X TE buffer; Sigma Aldrich) and blocking (10% normal goat serum, 1 h), sections were incubated for 12 h at 4 °C with antihuman αS (15G7 ALX-804-258-L001; 1:500, Enzo) or anti-phosphorylated (pSER129) αS (AB51253; 1:20,000; Abcam) in 10% normal goat serum. After washing with PBS, sections were incubated with the respective biotinylated secondary antibodies (1:200 in PBS; Vector Laboratories) and subsequently transferred into ABC solution (1:500 in PBS; VectaStain Elite Kit, Vector Laboratories) for 1 h and visualized with 3,3′-diaminobenzidin (DAB). Sections were then mounted, dried, and observed using a Zeiss AxioScan7 microscope (Karl Zeiss, Germany). For double-labeling the free-floating sections were blocked in 10% normal donkey serum and incubated overnight at 4°C with antibodies to hu αS (15G7, ALX-804-258-L001, 1:500; Enzo), anti-phosphorylated (pSER129) αS (AB51253; 1:2000; Abcam), tyrosine hydroxylase (TH) (AB152, 1:500; Millipore) or ubiquitin (AB 10201-2: 1:500; Abcam). This was followed by incubation with FITC-conjugated secondary antibodies (1:500 in PBS) for 4 h at RT. Confocal microscopy was conducted with a Zeiss LSM710 Confocal microscope (Karl Zeiss, Germany). Exposure time, gain, and light intensity were the same for all prepared slides. For confocal images of TH with 15G7-anti human-αS, each image was color-balanced. An ImageJ plug-in called “Colocalization highlighter” created a mask of either ubiquitin, pS129 αS overlapped with TH pixels.

### Assessment of striatal DA fiber integrity

25-µm-free-floating sections were rinsed in PBS (0.15 M NaCl, 0.1 M Tris-HCl, pH 7.5) and endogenous peroxidase activity quenched with 3% H_2_O_2_ in PBST for 30 min at RT. Unspecific protein binding was blocked with 10% normal donkey serum in PBS. Sections were incubated with rabbit TH (AB152, 1:500, Millipore) or VMAT2 (GTX89638, GeneTex; 1:2000) over night at 4 °C. Following 3 rinses with PBS, sections were incubated with secondary anti-rabbit (1:200, Dianova 711-065-152) in PBS containing 10% normal donkey serum, washed, and subsequently transferred into ABC solution (1:500 in PBS; Vectastain Elite Kit, Vector Laboratories) for 1 h and visualized with 3,3′-diaminobenzidin (DAB). Brain sections across a + 4.8 to +3.5 interaural range were chosen, referring to the Paxinos and Franklin mouse brain atlas^72^. Sections from all genotypes were simultaneously stained and digitized using constant imaging settings with identical light intensity, gain, and exposure time for all microscope slides and subsequent analyses were performed on a blinded basis. Images were converted to gray scale, and the mean gray value intensity was measured in the caudate/putamen (CPu) and in the adjacent corpus callosum (cc) to correct for signal background. Mean gray values were converted to uncalibrated optical density (UOD) using ImageJ 1.46r software (NIH). The UOD of TH signal in the CPu was calculated by the formula CPu_final(UOD)_ = CPu_(UOD) –_ cc_(UOD)_, similar to a previously published study. Threshold settings were set identically in the dorsal striatum for all sections. The integrated density of TH in the dorsal striatum was analyzed and plotted by an unbiased experimenter. To quantify the relative % neuronal loss, the TH+ neurons were exhaustively counted of the same sections used for fiber evaluation. All counting procedures and measurements were conducted at the NeuroTechnology Studio at Brigham and Women’s Hospital, that provided Zeiss Axioscan7 microscope, Zeiss LSM710 Confocal microscope, and Leica CM1950 Cryostat access and consultation on data acquisition and data analysis.

### Behavioral testing

#### Pole testing

Mice were placed head-up on top of a 50 cm vertical pole (all-thread metal rod) and tested for their ability to turn around and descend the pole (snout first). Recording began when the mouse initiated the turning movement and times to make a complete 180° turn and latency to reach the cage floor were recorded. Maximal duration time was set to 60 s to avoid exhaustion. The test consisted of 3 consecutive trials and average times were calculated for each mouse.

#### Gait scan analysis

To assess motor function and coordination in walking mice, automated gait analysis was performed using Treadscan (Cleversys Inc, Reston, VA). Gait patterns of 3–6 mo mice were measured for 25 s at a speed of 13 cm/s on a transparent running belt illuminated by a LED light and reflecting footprints captured by a video camera positioned underneath the walkway.

#### Rotarod

Motor coordination and motor skill learning were evaluated using an accelerating rotarod (Ugo Basile), and time spent on the rod was recorded. The first day consisted of a habituation trial at constant speed (4 rpm for 5 min), followed by two trials of 4–40 rpm progressive acceleration within 5 min for three consecutive days, including 2 trials each day. On these next there days, the mice were tested only on the accelerating trials (4–40 rpm, 5 min). An inter-trial pause of at least 1 h was applied to avoid fatigue and stress, and a maximum cutoff of 5 min was used. Motor coordination was evaluated by comparing the mean latency to fall over the three consecutive days between groups.

### Quantification and statistical analysis

Details regarding each statistical test, biological sample size (*n*) and *p* value can be found in the corresponding figure legends. All data are represented as mean ± SEM. SEM represents variance within a group. In all experiments, the genotypes can be found in the corresponding legends. Data were collected and processed side by side in randomized order for all experiments; most analyses were routinely performed blind to the conditions of the experiments. Unpaired, two-tailed t tests were used for comparison between two groups, with *p* < 0.05 considered significant. For all comparisons involving multiple variables, one-way or two-way ANOVA was performed followed by Tukey’s post hoc test for multiple comparison using *p* < 0.05 for significance. For all experiments, between 3–6 (biochemistry, histology) and 8–12 (behavior) animals per experiment were used, with the number per group stated in each figure legend. All statistical analyses were preformed using GraphPad Prism.

### Reporting summary

Further information on research design is available in the Nature Research Reporting Summary linked to this article.

### Supplementary information

Supplementary information

Reporting Summary

3D-mouse

WT-mouse

## Data Availability

All data generated (and commercially non-available tools used for analysis) during this study are included in this published article (and its Supplementary information files).
