# Author Correction: Generation of G51D and 3D mice reveals decreased α-synuclein tetramer-monomer ratios promote Parkinson’s disease phenotypes

**DOI:** 10.1038/s41531-024-00675-5

**Published:** 2024-03-12

**Authors:** Silke Nuber, Xiaoqun Zhang, Thomas D. McCaffery, Tim E. Moors, Marie-Alexandre Adom, Wolf N. Hahn, Dylan Martin, Maria Ericsson, Arati Tripathi, Ulf Dettmer, Per Svenningsson, Dennis J. Selkoe

**Affiliations:** 1 Ann Romney Center for Neurologic Diseases, Brigham and Women’s Hospital and Harvard Medical School, Boston, MA 02115 USA; 2 Neuro Svenningsson, Department of Clinical Neuroscience, Karolinska Institutet, 17176 Stockholm, Sweden; 3 Electron Microscopy Laboratory, Department of Cell Biology, Harvard Medical School, Boston, MA 02115 USA

**Keywords:** Parkinson's disease, Parkinson's disease

Correction to: *npj Parkinson’s Disease* 10.1038/s41531-024-00662-w, published online 29 February 2024

In this article, the author name Arati Tripathi was incorrectly written as Arathi Tripathi. The original article has been corrected.

