# Supplementary material for: Generation of G51D and 3D mice reveals decreased α-synuclein tetramer-monomer ratios promote Parkinson’s disease phenotypes
Source: NPJ Parkinsons Dis. 2024 Feb 29;10:47. doi: 10.1038/s41531-024-00662-w (PMC10904737; doi:10.1038/s41531-024-00662-w)
Supplement: Supplementary file 1 — Supplementary information [file 41531_2024_662_MOESM1_ESM.pdf]

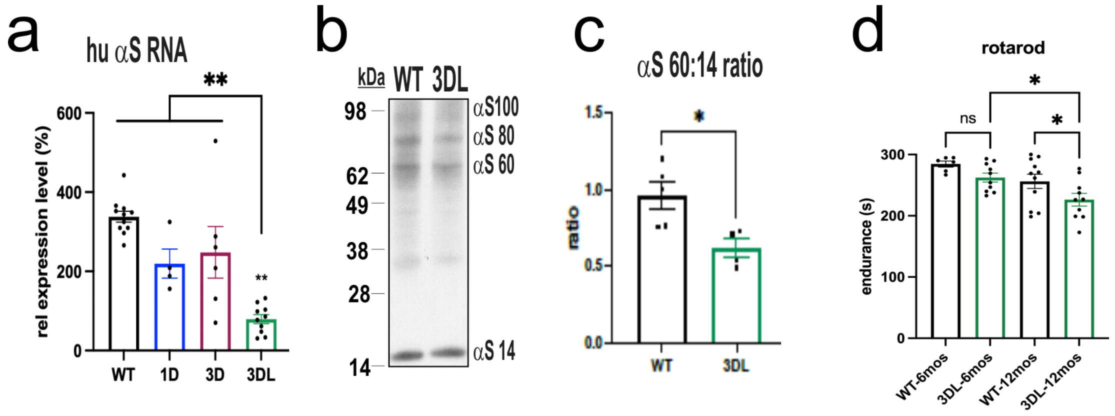

Supplementary Figure 1. (**a**) RNA analyses show similar expression of WT, 1D, 3D and a 2nd 3DL line with significantly lower expression level. (**b**) Representative WB of DSG cross-linked cortical samples reveals less αS T:M ratio in 3DL vs. WT, quantified in (**c**) . (**d**) Progressive deterioration between 6 and 12 mos in 3DL αS tg mice. *p<0.05, **p<0.01. Data are mean+/-SEM. One way ANOVA, post hoc Tukey.

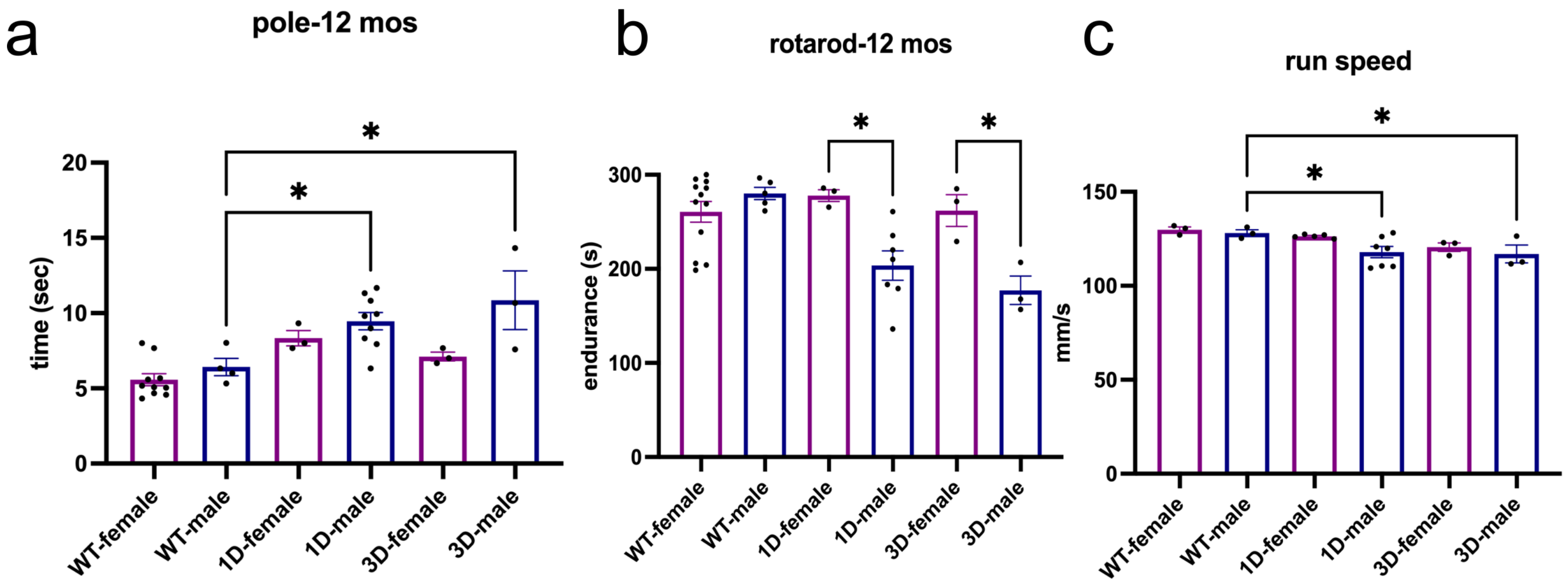

Supplementary Figure 2. Progressive motor deficits are more pronounced in male 1D and 3D αS tg mice. **a** Graph quantifies ability to turn and climb down a pole at 12 mos. **b** Graph quantifies motor and balancing skills on a 4-40 rpm accelerating rotarod. **c** Automated gait scan on a horizontal treadmill. Data are mean+/-SEM *$p<0.05$. Two-way ANOVA, post Tukey.

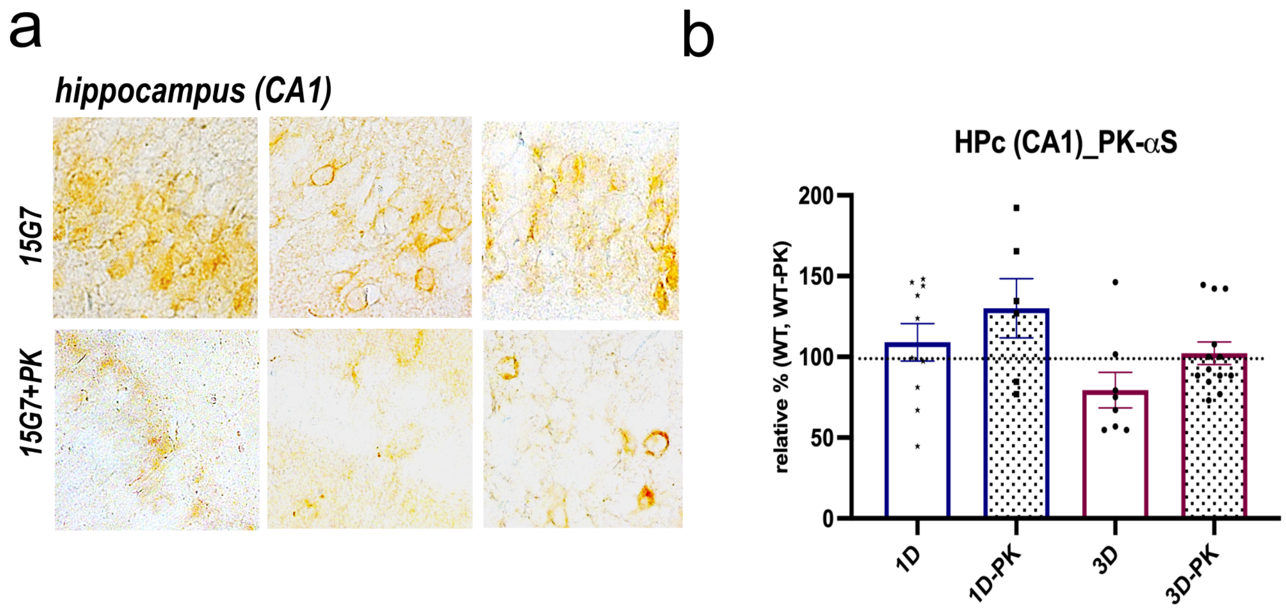

Supplementary Figure 3. 1D and 3D αS forms limited PK-resistant hippocampal CA1 inclusions. (**a**) Hippocampal sections were treated with proteinase K (PK) and the integrated optic densities with or without PK treatment (**b**) quantified as relative % against WT or WT-PK immunoreactivity. Data are mean+/- SEM. Two-way ANOVA, post Tukey.

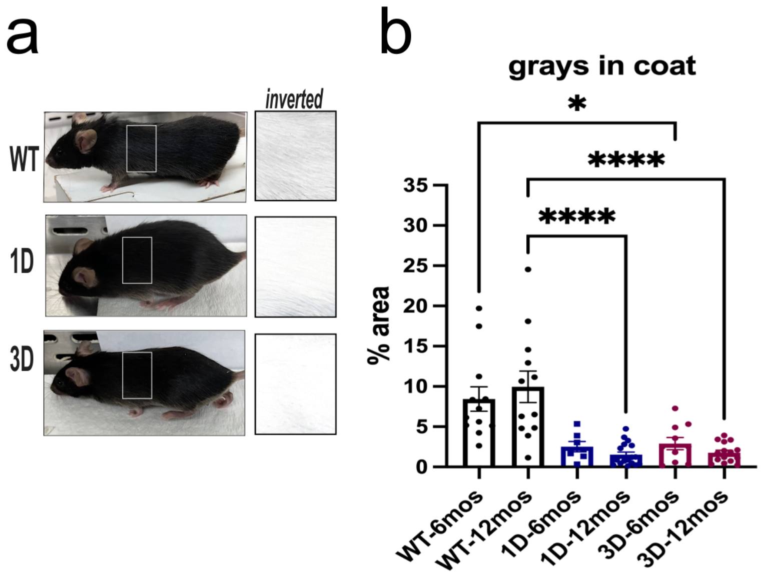

Supplementary Figure 4. Grays in the coat of WT, 1D, and 3D αS mutant mice. (**a**) Images were inverted (insets) for (**b**) quantification of relative % coverage of grays per field, using Image J software. *$p<0.05$, ****$p<0.0001$. Two-way ANOVA, post hoc Tukey.
